# Inflammation and cancer cell survival: TRAF2 as a key player

**DOI:** 10.1038/s41419-025-07609-w

**Published:** 2025-04-14

**Authors:** Adriana Albini, Luisa Di Paola, Giampiero Mei, Denisa Baci, Nicola Fusco, Giovanni Corso, Douglas Noonan

**Affiliations:** 1 European Institute of Oncology (IEO), Istituto di Ricovero e Cura a Carattere Scientifico (IRCCS), Milan, Italy; 2 Unit of Chemical-Physics Fundamentals in Chemical Engineering, Faculty Department of Science and Technology for Sustainable Development and One Health, Università Campus Bio-Medico, Rome, Italy; 3 Department of Experimental Medicine, University of Rome Tor Vergata, Rome, Italy; 4 Department of Biotechnology and Life Sciences, University of Insubria, Varese, Italy; 5 Molecular Cardiology Laboratory, IRCCS-Policlinico San Donato, Milan, Italy; 6 Department of Oncology and Hemato-Oncology, University of Milan, Milan, Italy; 7 Division of Breast Surgery, European Institute of Oncology (IEO), Istituto di Ricovero e Cura a Carattere Scientifico (IRCCS), Milan, Italy; 8 IRCCS MultiMedica, Milan, Italy

**Keywords:** Cancer microenvironment, Tumour biomarkers

## Abstract

TNF receptor-associated factor 2 (TRAF2) plays a crucial role in both physiological and pathological processes. It takes part in the regulation of cell survival and death, tissue regeneration, development, endoplasmic reticulum stress response, autophagy, homeostasis of the epithelial barrier and regulation of adaptive and innate immunity. Initially identified for its interaction with TNF receptor 2 (TNFR2), TRAF2 contains a TRAF domain that enables homo- and hetero-oligomerization, allowing it to interact with multiple receptors and signaling molecules. While best known for mediating TNFR1 and TNFR2 signaling, TRAF2 also modulates other receptor pathways, including MAPK, NF-κB, and Wnt/β-catenin cascades. By regulating NF-κB-inducing kinase (NIK), TRAF2 is a key activator of the alternative NF-κB pathway, linking it to inflammatory diseases, immune dysfunction, and tumorigenesis. In the innate immune system, TRAF2 influences macrophage differentiation, activation, and survival and stimulates natural killer cell cytotoxicity. In the adaptive immune system, it represses effector B- and T-cell activity while sustaining regulatory T-cell function, thus promoting immune suppression. The lack of fine-tuning of TRAF2 activity leads to excessive NF-kB activation, driving chronic inflammation and autoimmunity. Although TRAF2 can act as a tumor suppressor, it is predominantly described as a tumor promoter, as its expression has been correlated with increased metastatic potential and poorer prognosis in several types of cancer. Targeting TRAF2 or TRAF2-dependent signaling pathways might represent a promising anti-cancer therapeutic strategy.

## Facts

- TRAF2 activates the NF-κB pathway, driving inflammation, cell survival, and tumor progression while contributing to immune evasion and therapy resistance.
- TRAF2 stabilizes key proteins in the Wnt/β-catenin pathway, driving tumor growth and metastasis.
- TRAF2 promotes VEGF-driven tumor angiogenesis and supports immune suppression through Tregs and MDSCs.
- TRAF2 supports tumor survival during ER stress, aiding resistance to anti-cancer therapies.
- TRAF2 mostly acts as a tumor promoter in different cancer types and contexts.
- TRAF2 inhibition could enhance anti-cancer therapies and improve tumor killing.

## Open questions

- How can the structural dynamics of TRAF2, such as its monomer-trimer equilibrium and domain-specific interactions, be harnessed for drug development?
- Can TRAF2 expression or activity serve as a biomarker for predicting cancer prognosis or therapy response?

## Introduction

Inflammation plays a central role in different stages of cancer, including tumor development and malignant transformation [1]. TNF receptor-associated factors (TRAFs) are key mediators in the intricate interplay between cancer and inflammation, making them promising therapeutic targets. TRAFs belong to the TRAF family of signaling molecules that interact with a plethora of molecules thanks to their characteristic shape [2]. In complex with other proteins, TRAFs transduce signals upon activation of cytokine receptors, especially those belonging to the TNF receptors superfamily (TNFRs). TNFRs include death or decoy receptors [3], which bind to several types of ligands [4]. TNF-α, one of the major inflammatory cytokines, binds to TNF receptors 1 and 2 (TNFR1 and 2), triggering signaling pathways that regulate inflammation, immune responses, stress reactions, and apoptosis [5]. Despite sharing TNF-α as their primary ligand, TNFR1 and TNFR2 differ significantly in their structural features, signaling mechanisms, and biological outcomes. TNFR1 contains a death domain (DD) in its cytoplasmic region, which recruits DD-containing cytoplasmic proteins, such as TRADD and RIPK1. This leads to the activation of the transcription factor NF-kB, as well as apoptotic and necroptotic pathways [6–8]. In contrast, TNFR2 lacks a DD and predominantly activates non-canonical NF-κB and PI3K/Akt pathways, promoting cell survival, immune regulation, and tissue regeneration [9–11]. While TNFR1 is widely expressed across various cell types, TNFR2 expression is more restricted, mostly found in immune cells, endothelial cells, and certain cancer cells, where it plays a key role in inflammation and immune suppression [12].

TRAF2 and TRAF5 are structurally and functionally similar, sharing redundant roles in TNF-induced NF-κB activation. However, while TRAF2 is ubiquitously expressed, TRAF5 expression is limited to the lungs, thymus, spleen, and kidneys [13].

TRAF2, a 56 kDa trimeric protein, was initially identified for its interaction with the C-terminal portion of TNFR2 [14]. Structurally, TRAF2 consists of a globular C-terminal portion (TRAF-C) and an extended coiled-coil moiety (TRAF-N) comprising three long α-helices [15]. The combination of these two structurally different regions gives rise to the so-called “TRAF domain,” which is responsible for the assembly of the homo-oligomeric form [15], for the establishment and stabilization of the hetero trimer TRAF2:TRAF1 (with a 2:1 ratio) and for the interaction with other signaling proteins [3, 16, 17].

The monomer-trimer equilibrium plays a pivotal role in the control of TRAF2 functions [18], being also crucial for membrane binding [19]. In silico simulations suggest that the length of the coiled-coil region strongly influences the structural dynamics at the three interfaces present in the globular, C-terminal domain [20]. In particular, the reciprocal interaction of the long α-helices reduces the asymmetric motions of the three subunits, indicating strong communication between the two protein domains. Like other TRAF family members, TRAF2 also contains within the N-terminal region a predicted RING motif, displaying a ubiquitin E3 ligase activity connected to the coiled-coil section of the TRAF domain via five zinc finger motifs [15].

By linking the receptors to their downstream effectors, TRAF2 tightly regulates signaling pathways implied in many physiological and pathological processes, from inflammation to cancer [3]. However, the molecular mechanisms behind the TRAF2 multiple activities are still poorly understood due to the limited information about its molecular structure. The knowledge about TRAF2 dynamics and its role in membrane association and membrane vesicle trafficking is, in fact, generally limited to some TRAF2 fragments [18–23] because the equilibrium between TRAF2 monomeric and oligomeric forms and the presence of the long-coiled coil (TRAF-N) domain have hindered its purification. Despite these challenges, an expanding body of literature highlights this molecule’s pivotal role in unraveling the mechanisms underlying various physiological and pathological phenomena [7]. Under physiological conditions, TRAF2 modulates immune responses by supporting macrophage activation, T-cell differentiation, and the stability of regulatory T cells (Tregs) [24]. It also influences cell survival [25], maintains epithelial barrier integrity [26], and governs autophagy and the ER stress response [27–29]. TRAF2 enhances proinflammatory signaling, positioning it as a key driver of cancer-associated inflammation. An extensive review has been written by Siegmund et al. in 2022 on TRAF2 signaling and roles [3]. Our present work updates and expands upon key insights into TRAF2’s structural, molecular and mechanistic roles and its functions on the immune system. We elucidate how TRAF2’s signaling contributes to physiological functions as well as to cancer progression through direct mechanisms, inflammation, and the tumor microenvironment (TME), providing examples across cancer types. Additionally, we outline potential therapeutic strategies, including candidate inhibitors and combination approaches, and highlight future research directions to deepen the knowledge on TRAF2’s pivotal role in cancer biology.

## TRAF2 structure and function

### Insights on TRAF2 structural features and conformational dynamics

**Fig. 1:**
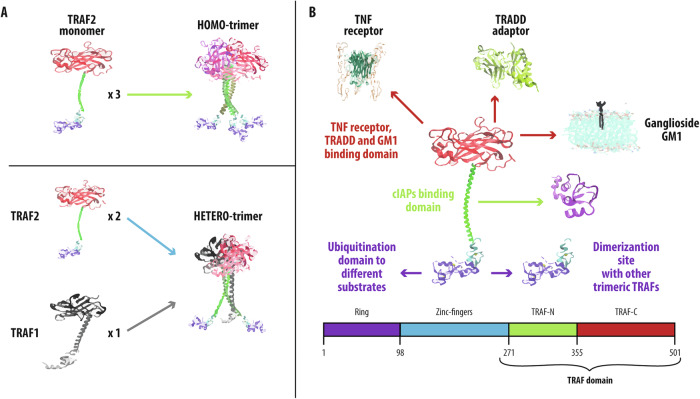
Structure-to-function relationship of TRAF2. **A** Self-oligomerization of TRAF2 (upper panel) and heterotrimer (TRAF2:TRAF1) formation (lower panel). The (partial) available structures of monomeric TRAF2 domains are represented in different colors: purple and cyan = RING domain + first zinc finger motif (PDB file: 3knv); green = TRAF-N (PDB: 3m06); red = TRAF-C (PDB: 1ca4). **B** The structure of selected partners of TRAF2 is shown (extracted from the following PDB files: receptor, 1tnr; TRADD, 1f3v; cIAPs, 3m0a). TRAF2 partners are correlated to the corresponding interacting regions of TRAF2 (red, green and purple arrows). At the bottom, the position of the four TRAF2 domains in the sequence is represented in the corresponding colors. The union of the TRAF-N and TRAF-C is referred to as the “TRAF domain,” following the traditional nomenclature reported in the literature.

The C-terminal portion (TRAF-C) and the coiled-coil tail (TRAF-N) are the most studied TRAF2 domains, regulating its oligomerization [18] and membrane binding [19]. These domains exhibit distinct structural and dynamic properties. TRAF-N has a typical elongated, sticky coiled-coil shape (Fig. 1) with a single secondary structure, while each TRAF-C subunit features a β-sandwich motif [4] and two monomer-monomer interfaces. Interactions at these interfaces, involving H-bonds and hydrophobic contacts, display dynamic behavior, enabling continuous subunit rearrangements in in silico simulations [30]. These movements, largely asymmetric, cause transient clustering of two monomers while the third remains independent, affecting protein surface shape and receptor peptide binding, as shown by molecular docking [18, 30]. Molecular dynamics simulations [20] further suggest that the coiled-coil section strongly influences C-terminal dynamics, indicating interdomain communication. Specifically, interactions among the three long α-helices reduce subunit asymmetry and surface roughness. Overall, these findings highlight the crucial role of TRAF2’s oligomeric structure in receptor and ligand recognition, as well as interaction regulation.

### Overview of TRAF2 signaling pathways

Germline- and cell-specific TRAF knock-out models have provided insight into the essential roles of TRAFs in several signal transduction cascades [30]. TRAF2 is a principal mediator of TNF-α signaling, acting downstream of its receptors, TNFR1 and 2 [31], thus triggering the activation of NF-kB, the major transcription factor of inflammatory molecules [7]. Mitogen-activated protein kinases (MAPKs), such as c-Jun N-terminal kinases (JNKs), are also activated, inhibiting apoptosis and promoting cell survival and inflammation [17]. Beyond its role in cytokine signaling, TRAF2 controls key cellular processes through ubiquitination of many substrates and, in this way, regulates their stability and, thus, their functions [32]. TRAF2 is also the target of ubiquitination/deubiquitination, which, by regulating its subcellular localization, dictates the cell fate between survival and death [21]. The activation of NF-κB relies on the recruitment of TRAF2 to plasma membrane lipid rafts, a process disrupted when TRAF2 is ubiquitinated, thereby impairing its signaling ability [33].

### TRAF2-mediated TNFR1 and TNFR2 signaling pathways

**Fig. 2:**
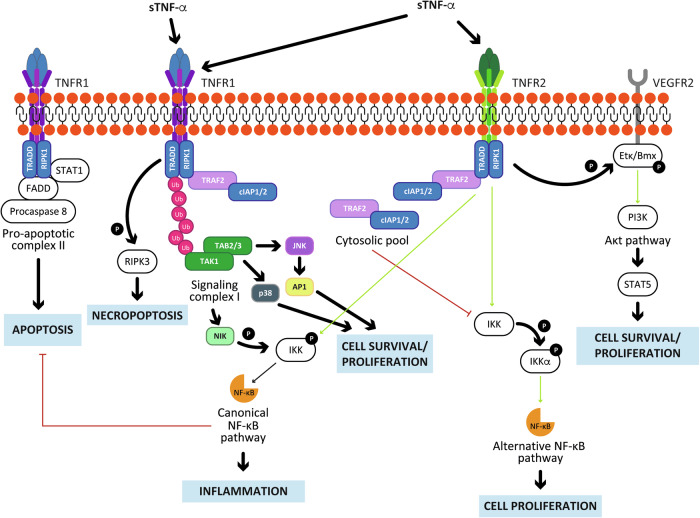
TRAF2 role in the TNFR1 and TNFR2 signaling pathways. TNFR1 is activated either by mTNF-α or sTNF-α and recruits TRADD, RIPK1, TRAF2 and cIAP1/2, forming Complex I. cIAP1/2 ubiquitinates RIPK1 creating a platform for the recruitment of TAB2/3 and TAK1 which, in turn, activate JNK, AP-1 and p38 resulting in cell survival and activates NIK, which triggers NF-kB activation promoting inflammation. When RIPK1 is deubiquitinated, cell death is triggered either through apoptosis, which is activated by the formation of the FADD/pro-caspase 8 complex, or through necroptosis, which is induced by the interaction between RIPK1 and RIPK3. TNFR2 is activated by mTNF-α and recruits TRAF2 and cIAP1/2 and, in this way, activates the noncanonical NF-kB pathway and cell proliferation. TNFR2 also activates Etk/Bmx, which, by forming a complex with VEGFR2, activates the PI3K/Akt/STAT5 axis.

TRAF2 acts downstream of TNFR1, which is involved in innate and adaptive immunity responses, inflammation, apoptosis, and necroptosis (Fig. 2) [34]. TNF-α triggers TNFR1 activation in either membrane-bound (mTNF-α) or soluble (sTNF-α) forms and initiates TNFR1 trimerization and the assembly of complex I, which recruits RIPK1 and TRADD, both containing a death domain (DD) [35]. There is further recruitment of the ubiquitin ligases cellular inhibitors of apoptosis proteins 1 and 2 (cIAP1 and cIAP2), which stabilize RIPK1 [36].

RIPK1 then recruits TAB1/2 and MAP3K, activating the kinase TAK1, which in turn triggers NF-κB, p38, AP-1, and JNK signaling pathways, promoting inflammation, cell survival, and proliferation [37–40]. TAK1 also activates the kinase NIK that, by phosphorylating IKK, activates the alternative NF-kB pathway [41].

When TNFRs are unstimulated, the cIAP–TRAF2 complex inhibits the alternative NF-κB pathway by promoting the degradation of NIK [42]. If complex I fails to assemble, complex II, composed of FADD, TRADD, and STAT1, forms and mediates TNF-α-induced apoptosis via TNFR1 through activation of caspases 8 and 3 [43]. TRAF2 prevents excessive apoptosis by facilitating the proteasomal degradation of activated caspase-8 [44]. If caspase activity is insufficient, an alternative complex II forms with RIPK1 and RIPK3, activating the pseudokinase MLKL and leading to necroptotic cell death. This pathway eliminates damaged or infected cells, while its dysregulation can result in uncontrolled cell death or viral infection [45].

TNFR2, primarily expressed in immune cells [46], lacks a DD and promotes anti-inflammatory functions, neuroprotection and remyelination [9]. Upon activation by TNF-α, TNFR2 weakly associates with the TRAF2-cIAP1–cIAP2 complex, which activates noncanonical NF-κB signaling by inhibiting NIK degradation [10]. TNFR2 also enhances cell survival and proliferation by forming a complex with the tyrosine kinase Etk/Bmx and VEGFR2, activating the PI3K/Akt pathway and downstream STAT5 signaling [11].

A schematic representation of the TNFR1 and TNFR2 pathways mediated by TRAF2 is depicted in Fig. 2.

### Roles of the TRADD–TRAF2 interaction

TRADD is essential in determining the downstream effects of TNF signaling by connecting TRAF2 to TNFR1 and recruiting RIPK and FADD. The high-affinity interaction between TRAF2 and TRADD ensures the TNFR1-dependent activation of NF-κB and JNK, resulting in apoptosis inhibition and inflammatory response [47]. Conversely, TRADD binds to FADD to trigger the apoptotic cascade when NF-κB activation is inhibited. TRADD also binds RIPK3 and promotes its activation, eliciting TNF-mediated necroptotic signal transduction [48]. The rapid NF-κB response upon TNFR1 activation prevails over the slow activation of caspase 8 and apoptosis, which, on the contrary, is enhanced in situations of TRADD deficiency [49]. TRADD also inhibits the ubiquitination by TRAF2/cIAP1/cIAP2 of the autophagy mediator, beclin 1, thereby reducing autophagy [27, 50].

### TRAF2 in the regulation of the Wnt-β-catenin signaling pathways

**Fig. 3:**
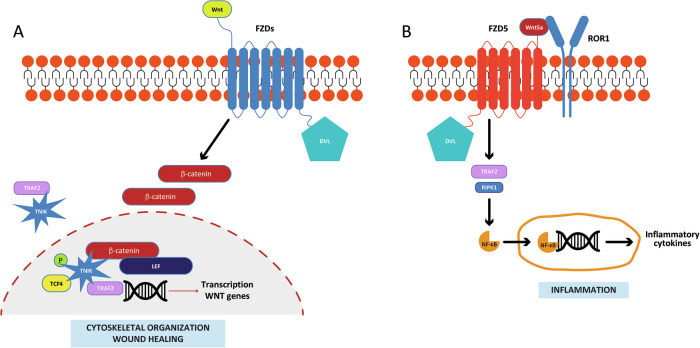
TRAF2 role in the canonical and noncanonical Wnt signaling pathways. **A** In the canonical pathway, Wnt activates its receptor, FZD, and results in the recruitment of the scaffold protein, DVL, and to the cytoplasmic accumulation of β-catenin. TRAF2 participates in the TNIK-dependent activation of TCF-4/LEF, which forms a complex with β-catenin and activates the transcription of Wnt target genes involved in cytoskeletal organization and wound healing. **B** The noncanonical pathway is triggered by the interaction of Wnt5a to FZD5 and ROR1, which promotes the formation of the TRAF2/RIPK1 complex and results in the activation of NF-kB and the promotion of inflammation.

TRAF2 regulates canonical Wnt-β-catenin signaling pathways (Fig. 3). By stimulating its G-protein coupled receptor, Frizzled (FZD), Wnt triggers the recruitment of the scaffold protein Disheveled (DVL), leading to cytosolic accumulation of β-catenin and its subsequent translocation to the nucleus, where it activates the transcription factors TCF (T-cell factor)/lymphoid enhancing factor (LEF), driving Wnt target genes transcription [51]. One of the pathways triggered by Wnt involves the kinase TNIK (TRAF2 and Nck-interacting kinase), which interacts with TRAF2 and β-catenin and binds, phosphorylates and activates TCF4 [52]. A recent study demonstrated that the engagement of TNIK/β-catenin in cytoplasm and nucleus is TRAF2-dependent and regulates cytoskeletal organization [53] (Fig. 3A). By sustaining the Wnt-β-catenin signaling [54], TRAF2 may also contribute to wound healing. TRAF2 modulates the β-catenin-independent noncanonical Wnt signaling pathway, mediated by Wnt5a through the complex between the receptor tyrosine kinase-like orphan receptor 1 (ROR1), Frizzled 5 (FZD5) and DVL [55, 56]. TRAF2 was shown to be essential for the Wnt5a-driven inflammatory cytokine secretion via NF-κB activation (Fig. 3B) [57].

## TRAF2 physiological functions

### TRAF2 roles in ER stress-induced autophagy, cell death and epithelial barrier homeostasis

TRAF2 plays a critical role in the ER stress pathway by interacting with the stress sensor inositol-requiring enzyme 1 (IRE1), activating JNK signaling. TRAF2 can either prevent apoptosis triggered by excessive ER stress [58] or, through IRE1, can also induce cell death, activating caspase-12 [28, 59]. Additionally, TRAF2 promotes autophagy, including mitophagy in cardiac myocytes, which is crucial for cytoprotection during ischemia-reperfusion injury [29]. TRAF2 supports epithelial barrier integrity by regulating reactive oxygen species (ROS) production, JNK signaling, and autophagy in response to oxidative stress. TRAF2 downregulation attenuates the TNF-α/JNK pathway, enhancing E-cadherin expression, which maintains the apical–basal polarity in differentiated epithelia and strengthens epithelial barrier integrity [26]. Furthermore, TRAF2 supports intestinal cell protection, regulates epithelial–mesenchymal transition (EMT), promotes wound healing [60] and influences cell cycle progression [61].

**Fig. 4:**
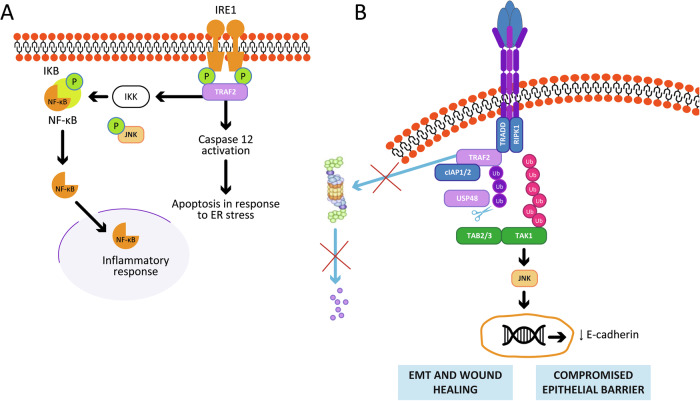
TRAF2 roles in ER stress response and epithelial barrier homeostasis. **A** TRAF2 interacts with the cytoplasmic portion of IRE1 and promotes the activation of JNK and NF-kB, thus inducing an inflammatory response. Conversely, TRAF2 induces the activation of caspase 12 and the apoptotic process. **B** Downstream of TNFR1, USP48 promotes the stabilization of TRAF2, leading to the activation of JNK and downregulation of E-cadherin. In this way, TRAF2 contributes to the decreased epithelial barrier.

Figure 4 illustrates TRAF2’s roles in ER stress and epithelial barrier homeostasis.

### TRAF2 roles in inflammatory and immune system cells

**Fig. 5:**
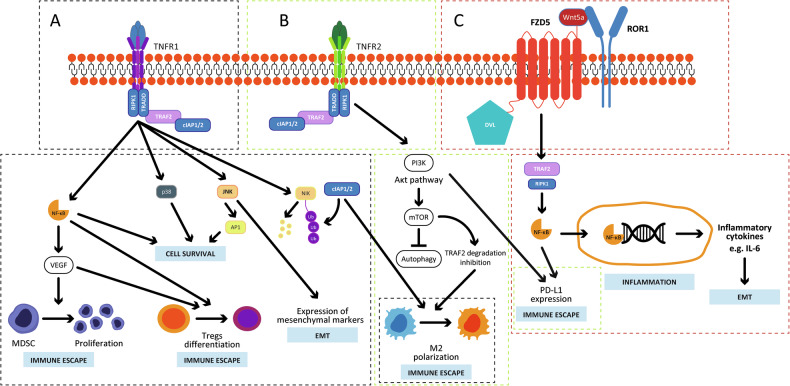
TRAF2 roles in cancer. **A** TRAF2 forms a complex downstream of TNFR1 with RIPK1, TRADD, and cIAP1/2, which inhibits apoptosis and activates NF-kB, JNK and p38. NF-kB, in turn, increases the expression of VEGF, thus sustaining angiogenesis. Also, both VEGF and NF-kB promote the differentiation of Tregs, thus sustaining immune escape. VEGF contributes to immune suppression by stimulating the proliferation of MDSCs. p38 and JNK promotes cell survival, and, in addition, JNK sustains EMT and, therefore, cancer aggressiveness. By promoting NIK degradation, the TRAF2-mediated TNFR1 signaling seems to play a role in the polarization of macrophages towards an M2 pro-tumoral phenotype. **B** TRAF2 stimulates cancer aggressiveness by activating the PI3K/Akt/mTOR signaling pathway downstream of TNFR2. This pathway inhibits autophagy and, by suppressing TRAF2 degradation, promotes the M2 macrophage polarization. **C** TRAF2 sustains the noncanonical Wnt signaling mediated by Wnt5a, which activates NF-kB and triggers inflammation. The dotted squares indicate the molecules or cellular events activated by each of the three receptors (purple dotted squares: TNFR1 pathway; green dotted squares: TNFR2 pathway; red dotted squares: Wnt5a pathway). DVL Disheveled, EMT epithelial–mesenchymal transition, FZD Frizzled.

TRAF2 plays a critical role in maintaining immune system homeostasis by regulating the activation, proliferation, and differentiation of immune cells, such as macrophages, NK cells, T and B cells, Tregs, Th17 cells, dendritic cells (DCs) and mesenchymal stem cells (MSCs) [24]. TRAF2 degradation by cIAP1 is necessary for monocyte differentiation into active macrophages [62], while its signaling via TNFR1/2 promotes inflammatory and survival pathways in macrophages (Fig. 5) [22]. Moreover, TRAF2 supports type I IFN expression either in macrophages or DCs and is, therefore, crucial for infection defense [63]. TRAF2 enhances NF-κB signaling, mediating processes like Fas expression in NK cells [64], TNFR2-driven IL-2 production, and, through the PI3K/Akt pathway, supports the expansion and stability of Tregs and suppression of Th17 [65, 66]. TRAF2 can also influence MSC immunosuppressive functions through its activity in supporting the TNF-α signaling pathway [67] and modulating regulatory B cell (Breg) activity [68]. Also, TRAF2 is involved in the activation of NF-kB downstream of CD40 and sustains B-cell activation and the isotype switch [69]. It has also been shown that TRAF2 inhibits DC cell apoptosis induced by TNF-α by activating NF-kB and the expression of IL-12 and anti-apoptotic genes [70].

The functions of TRAF2 in the immune system are summarized in Table 1 [32, 62–75].

**Table 1:** TRAF2 functions in the innate and adaptive immune systems.

Target cell type	TRAF2 functions
**Innate immunity**	
Macrophages	• Hinders the differentiation of monocytes into fully active macrophages [62] • Promotes inflammatory activities and controls macrophage activation, survival and death [71] • Induces type I interferon and immune responses against infections [63]
DC	• Regulates DC functions [72] • Sustains type I IFN induction and immune responses against infections [63] • Sustains the activation of NF-kB and the expression of IL-12 and anti-apoptotic genes [70]
MDSCs	• Enhances the immune suppressive properties [73]
NK cells	• Enhances NF-kB activation, which increases the expression of Fas, thus promoting cytotoxic activity [64]
**Adaptive immunity**	
CD8 + T cells	• It is essential for the activation of TNFR1 pro-survival signaling in activated CD8 + T cells [74]
Memory T cells	• Activation and survival [75]
Th17	• Regulates the differentiation [65, 66]
Tregs	• Promotes the differentiation [65, 66] • Sustains the phenotypic stability, proliferation, activation and suppressive activity [66]
B cells	• Negatively regulates the survival of B cells [32] • Sustains B-cell activation [69] • Regulates B-cell CD40 function in T-dependent antibody responses [72]
Bregs	• Modulates the secretion of inflammatory cytokines [68]
**Stem cells with immunomodulatory effects**	
MSCs	• Enhances immunosuppression by repressing the proliferation and functions of Teffs and Beffs and stimulating the activities of immune suppressive cells [67]

## TRAF2 role in cancer

### TRAF2 expression in cancer

*TRAF2* has been identified as an oncogene frequently mutated in cancer [76]. TRAF2 represents a negative prognostic factor, which is required for the malignant phenotype, anchorage-independent growth and increased resistance to chemotherapy and radiotherapy of several cancers [77]. Suppression of TRAF2 in these cancer cells results in NF-κB downregulation [76] and apoptosis [78]. Among the rare paradoxical effects of TRAF2, TRAF2 inactivating mutations increase alternative NF-κB pathway activation in mantle cell lymphoma, diffuse large B-cell lymphoma and multiple myeloma [79, 80].

### Effect of TRAF2 in cancer cells and the establishment of an immunosuppressed tumor microenvironment

TNF and its receptors are essential components of the tumor microenvironment (TME). TNFR1 drives inflammation, apoptosis, or necrosis, depending on the cell type, while TNFR2 promotes cell proliferation. TNFR1’s role in the TME is well-studied, whereas TNFR2 has only recently gained attention. While TNFR2 is rarely expressed in normal tissues, it is frequently overexpressed in cancer cells and other TME cellular components [81]. In cancers, TRAF2 overexpression inhibits the apoptotic process while promoting angiogenesis, tumorigenesis [82, 83] and tumor progression [84].

TRAF2 has a prominent role in activating NF-kB, whose activity is detrimental in the context of chronic inflammation or tumorigenesis [85]. Persistent NF-kB activation by TRAF2-dependent signaling pathways triggers the production of proinflammatory cytokines, which contribute to the survival and progression of cancer cells and are a hallmark of many inflammatory diseases. Inflammation promotes tumorigenesis, suppresses anti-tumor immunity, and drives an immunosuppressive TME [86, 87]. Recruited immune cells release cytokines and reactive oxygen species (ROS), which induce DNA damage, impair repair, and contribute to cellular transformation and tumorigenesis [88]. TRAF2 seems to promote malignant transformation by driving EMT [89–91] as its degradation increases the expression of epithelial markers, such as E-cadherin, and decreased expression of the mesenchymal marker, vimentin [84]. In this way, TRAF2 also disrupts the E-cadherin-β-catenin complex, leading to uncontrolled β-catenin signaling. Consequently, cancer cells lose adhesion, gain motility, and exhibit increased infiltration and metastatic potentials [92–94]. The TRAF2-dependent EMT induction may result from Wnt5a-mediated NF-κB activation and subsequent IL-6 secretion [47, 95]. Since persistent stress conditions typically characterize the TME, the TRAF2-sustained ER stress response aids cancer cells and other TME components adapt to ER stress induced by chemotherapeutic agents, thus avoiding apoptosis and promoting chemoresistance [96].

TRAF2 is recruited by IRE1, where it forms a complex that activates the NF-κB pathway and the subsequent transcription of anti-apoptotic genes, which help tumor cells resist ER stress-induced cell death [97]. By interacting with IRE1, TRAF2 also leads to prolonged activation of the JNK pathway [98]. By inducing ER-stress-mediated autophagy and EMT, JNK promotes aggressiveness and resistance to anti-tumoral drugs [99].

Since TRAF2 promotes the expansion and tumor infiltration of immune suppressive cells, favoring immune escape [100], it might also shape the TME immunological characteristics. Considering the activity of TRAF2 in keeping the balance between Tregs and effector T cells (Teffs) [46], TRAF2 could display a role in facilitating cancer immune evasion. It has been shown that TRAF2-dependent inhibition of apoptosis downstream of TNFRs is exploited by several cancers to escape from the T-cell killing [3]. TRAF2 also seems to induce VEGF expression that, in turn, besides promoting angiogenesis [100], induces immune tolerance by suppressing T-cell and APCs functions and activating Tregs and MDSCs [101]. Moreover, it is possible to suggest a role of the TRAF2-dependent TNFR2 signaling in the protection of MDSC from apoptosis and their accumulation during tumor growth [102]. TRAF2 could have a role in the development and aggressiveness of cancer cells by influencing the differentiation into Th17 cells [103, 104], which exerts immunosuppressive functions and stimulates angiogenesis by secreting IL-17 [105].

### TRAF2 role in specific cancers

In **gastric cancer** cell lines, TRAF2 silencing diminished the proliferation, migration and invasion [106]. Of note, in hereditary diffuse gastric cancer, the role of TRAF2 in promoting EMT could be particularly important since this tumor displays E-cadherin gene (*CDH1*) germline [92, 93] or somatic mutations, which correlate with increased aggressiveness and poor prognosis [94]. **In hepatocellular carcinoma (HCC)**, the ubiquitin-specific peptidase 24 (USP24) deubiquitinates and stabilizes TRAF2, which, in turn, promotes cell proliferation and metastasis [107, 108]. USP24 expression in HCC positively correlated with the expression of the immune evasion molecule, programmed cell death ligand 1 (PD-L1) [107]. Therefore, TRAF2 inhibition might be crucial for increasing the effectiveness of immunotherapy in HCC. In HCC, TRAF2 also promotes tumor growth by preventing ROS production, mitochondrial dysfunction [109] and autophagy [110] and contributes to drug resistance by supporting ER stress response [111]. By promoting the activation of the noncanonical NF-kB pathway, TRAF2 supports cell proliferation [110, 112], resistance to apoptosis in both hepatitis B virus-derived HCC and spontaneous HCC [113] and macrophage polarization toward a pro-tumoral M2 phenotype [114]. **In non-small-cell lung cancer cells (NSCLC)**, TRAF2 confers resistance to TNF-mediated apoptosis [76, 82] and resistance to therapies [115, 116]. In **ovarian cancer** cells the DUB, UCHL3, is responsible for activating NF-κB signaling by stabilizing TRAF2 thus facilitating tumorigenesis [117]. Also, TRAF2 overexpression was described to be associated with resistance to radiotherapy in **nasopharyngeal carcinoma** cells [118]. Moreover, TRAF2 has been described as being a dominant oncogenic driver of **colon cancer** development supporting the Wnt-β-catenin signaling pathway [53]. Besides this, TRAF2 seems to mediate EMT induced by low oxygen levels in this cancer type [119].

In **breast cancer** (BC), TRAF2 upregulation increased the migration of tumor cells and supported osteolytic metastasis by enhancing osteoclastogenesis. The authors suggested that the aggressive behavior and bone tropism of BC cells are, at least in part, dependent on the TRAF2/NF-kB axis [120]. Moreover, TRAF2 seems to promote BC cells’ resistance to cell-detachment-induced apoptosis (anoikis) [121]. Given the importance of Th17 cells in BC [122], TRAF2 may enhance BC aggressiveness by promoting Th17 cell differentiation. In **prostate cancer** (PCa) cell lines, it has been shown that TRAF2 participates in the PI3K/Akt pathway activation, inhibiting apoptosis and promoting cell proliferation, invasion, and migration [123]. In **melanoma** cells, findings suggest that TRAF2 sustains the downstream signaling of IL-17, increasing aggressiveness [124]. Xu et al. demonstrated that, by inhibiting autophagy, TRAF2 promotes the macrophage polarization towards the M2 pro-tumoral phenotype and their infiltration into the tumor, alongside angiogenesis and cancer progression in **clear cell renal carcinoma** [83]. In **B-cell lymphoma**, the potential role of inhibiting TRAF2 to increase the response toward chemotherapeutics has been suggested [125]. A recent study also suggested the role of the TNIK/TRAF2 complex in promoting **leukemia** progression [126].

TRAF2 seems to be required for tumor growth promotion by inhibiting apoptosis, promoting a malignant phenotype through EMT, regulating the sensitivity of certain cancer cells to chemotherapy and radiotherapy, and inducing immune suppression. Although evidence indicates that TRAF2, by promoting the activation of NF-κB, enhances the CD8+-related anti-tumor immune response [127], hints to double-edged sword effects of TRAF2 hyperactivation are rare, indicating it as mostly pro-cancerogenic.

Figure 5 schematically represents the TRAF2-mediated pathways involved in the proliferation and aggressiveness of cancer cells, and Table 2 describes the role of TRAF2 in the different types of cancer [53, 76, 79, 80, 82, 83, 92, 93, 103, 105, 107, 109–113, 115–121, 123–126].

**Table 2:** The role of TRAF2 as tumor promoter or tumor suppressor in different types of cancer.

Cancer type	TRAF2 activity/role
Gastric cancer	• Increases proliferation, migration and invasion [121] • Has a potential role in promoting EMT by downregulating E-cadherin
Hepatocellular carcinoma	• Promotes the Wnt-β-catenin signaling, which activates the PI3K/Akt and NF-κB signaling pathways promoting cell proliferation and metastasis [107, 108] • Supports the canonical NF-kB pathway, enhancing resistance to apoptosis [113] • It might play an important role in the resistance to immunotherapy by increasing PD-L1 expression • Prevents ROS production and mitochondrial dysfunction, protecting against cell cycle arrest and senescence [109] • By impairing NIK function, the complex cIAP–TRAF2 activates the noncanonical NF-κB pathway and supports cell proliferation [110, 112], resistance to apoptosis in both hepatitis B virus-derived HCC and spontaneous HCC [113] and macrophage polarization toward a pro-tumoral M2 phenotype thus contributing to cancer progression [114] • Activates mTOR, suppresses autophagy and promotes cell survival and proliferation by [110] • Contributes to drug resistance supporting ER stress response [111]
Non-small-cell lung cancer	• Enhances NF-κB activation, conferring resistance to TNF-mediated apoptosis [76, 82, 121] • Promotes cancer cell proliferation and potentially serves as a target to sensitize cells to argon-helium cryoablation [115] and radiotherapy [116]
Ovarian Cancer	• Enhances NF-κB activation, leading to tumorigenesis [117]
Nasopharyngeal carcinoma	• Induces resistance to radiotherapy [118]
Colon Cancer	• Stabilizes the β-catenin-TNIK-TCF4 complex, activating the expression of Wnt target genes, which drive tumor development [53] • Leads to JNK activation, which promotes EMT, especially under low oxygen conditions [119]
Breast cancer	• Increases tumor cell migration and promotes bone metastasis via osteoclastogenesis through NF-κB activation [120] • By interacting with focal adhesion kinase (FAK), TRAF2 seems to promote BC cell resistance to cell-detachment-induced apoptosis (anoikis) • TRAF2 may contribute to Th17 cell differentiation and cancer progression
Prostate cancer	• Activates PI3K/Akt, inhibiting apoptosis and promoting proliferation, migration and invasion [123]
Melanoma	• Sustains the downstream signaling of IL-17, resulting in the induction of proliferation and invasion [124]
Clear cell renal cell carcinoma	• Promotes M2 macrophage polarization and infiltration into tumors, aiding cancer progression and angiogenesis [83]
B-cell lymphoma	• Increases resistance to chemotherapeutics [125]
Leukemia	• The TNIK/TRAF2 complex activates Wnt signaling downstream of CD27, promoting tumor progression [126]
Mantle cell lymphoma Diffuse large B-cell lymphoma Multiple myeloma	• TRAF2 acts as a tumor suppressor [79, 80]

## Therapeutic strategies targeting TRAF2 or TRAF2-interacting molecules

Modulating TRAF2 expression or activity can influence downstream TNFR signaling and is emerging as a promising cancer therapy target due to its pro-tumoral role. However, effective TRAF2 inhibition is challenging, as it interacts with multiple binding partners and pathways. Researchers are investigating natural and synthetic molecules that directly target TRAF2 or its interacting partners as potential anti-cancer strategies.

In IFNγ-resistant melanoma cells, TRAF2 and cIAP1 were identified as key factors increasing sensitivity to CD8 + T-cell mediated killing by lowering the TNF cytotoxicity threshold and, therefore, increasing immunotherapy efficacy. This study showed that TRAF2 loss enhanced the efficacy of cIAP1/2 inhibition by the Smac/DIABLO mimetic birinapant. Combined TRAF2 and cIAP1/2 inhibition improved immune checkpoint blockade (ICB) efficacy in mouse cancer models [128]. Antagonists of cIAPs, which also inhibit TRAF2, are being evaluated as anti-cancer agents in clinical trials but have shown limited efficacy [129]. Since TNFRs can deplete cytosolic TRAF2 [34], their modulation could act as selective inhibitors of the cytosolic functions of TRAF2. Strategies that target TNFR2 seem to be of particular interest due to its expression being much higher in cancer cells and other TME cellular components [81]. There is an ongoing phase I/II clinical trial utilizing BI-1808, an anti-TNFR2 fully human antibody, as a single agent or in combination with immunotherapies in subjects with advanced cancers. BI-1808 was able to deplete the TME of Tregs, modulate MDSCs and promote CD8 + T-cell expansion [130]. According to these results, another anti-TNFR2 antibody, APX601, was also able to reverse the immune suppressive characteristics of the TME [131]. Eriodictyol, which is a flavonoid belonging to the subclass of flavanones, was able to induce apoptosis of tumor cells by enhancing TNFR1 expression and was able to reduce experimentally induced lung metastasis in vivo [132].

Besides this, TRAF2 also displays functions that are independent of TNFRs and cIAPs, suggesting that direct TRAF2 inhibitors might offer additional therapeutic benefits, especially in combination with ICB. TRAF2 silencing, through small interfering RNA, partially reversed radioresistance and significantly suppressed glioblastoma cell growth [116]. TRAF2-silencing also attenuated in vitro the migration and invasion capacities of gastric cancer cells [133]. TRAF2 ablation, besides boosting the classical NF-κB pathway downstream of TNFRs and redirecting TNF signaling favoring apoptosis, has also been suggested as a tool to increase the susceptibility of tumors to immunotherapy by counteracting ICB [3]. A meta-analysis of over 1000 ICB-treated patients found that TRAF2 loss correlated with a positive clinical response, suggesting that targeting TRAF2 could enhance ICB efficacy [134]. Liquidambaric acid (LDA), a pentacyclic triterpenoid, is a TRAF2-interacting molecule with potential anti-tumoral effects, shown to inhibit colon cancer growth. LDA selectively blocks β-catenin–TRAF2 interaction without affecting others, highlighting its potential for targeted TRAF2 inhibition [53]. An LDA analog selectively targeted TRAF2 with higher binding affinity than LDA, inhibiting Wnt and TNF signaling and hindering the growth of colon cancer cells in xenograft mice [106]. Peptidomimetics targeting the TRADD-TRAF2 interaction have been identified [135], but their usefulness to inhibit TRAF2 functions requires further investigation.

Additionally, TNF-like weak inducer of apoptosis (TWEAK), a ligand of Fn14, also makes cancer cells more vulnerable to TNF-mediated killing, supporting Fn14’s role in depleting cytosolic TRAF2-cIAP1/2 complexes [128]. Vince et al. [136] found that TWEAK-FN14 signaling leads to the lysosomal degradation of the cIAP1-TRAF2 complex, sensitizing tumor cells to TNF-α. Michaelson et al. [137] developed an Fn14 agonistic antibody that inhibited growth in approximately 50% of a tested panel of 38 human tumor cell lines.

CXC195 is a tetramethylpyrazine analog that induced apoptosis of a bladder cancer cell line by inducing mitochondrial dysfunction and ER stress-induced activation of JNK through the formation of an IRE1–TRAF2–ASK1 complex [138]. Verproside, a compound extracted from *Veronica* species, exhibits cytostatic activity by blocking the TNF/NF-kB signaling in rhabdomyosarcoma cell lines, and it seems that this action is due to its interaction with the TRAF2-TRADD complex [139]. TNIK inhibitors seem to be promising anticancer agents since they interfere with the TRAF2 activity of promoting the Wnt-β-catenin signaling. A TNIK inhibitor, NCB-0846, due to its ability to block the EMT process in lung cancer cells, represents a potentially promising approach for the prevention of metastasis [140]. NCB-0846 was also able to sensitize TNIK-overexpressing lung squamous cell carcinoma to radiotherapy in in vitro and in vivo models [141] and to hinder papillary thyroid carcinoma progression by inhibiting cytoskeletal remodeling [142]. A recent study showed that OBD9, an oxetane derivative of the benzimidazole mebendazole with TNIK inhibitory activity, exhibits strong anticancer activity in various cancer cells, particularly colorectal cancer, by inducing TNIK autophagic degradation and blocking TCF4/β-catenin-mediated gene expression [143]. It has been shown that wogonoside, a flavonoid compound, inactivated NF-κB signaling by decreasing TRAF2/4 expression. Consequently, Wogonoside inhibited Twist, a transcription factor driving EMT, thereby downregulating MMP-9, MMP-2, vimentin, and CD44v6 expression in TNF-α-induced triple-negative breast cancer (TNBC) cells [144]. This article suggests wogonoside as a potential TNBC therapy and supports TRAF2’s role in EMT, highlighting its potential as a therapeutic target in epithelial cancers. Structure-based computational screening identified NF023, a suramin analog, as a candidate for modulating NF-κB by disrupting the cIAP2-TRAF2 interaction, potentially affecting cancer cell survival [23].

Fang et al. showed that Xinfeng Capsule is able to inhibit NF-kB activation by downregulating TRAF2 [145]. Therefore, it is possible to speculate the use of this medicinal plant blend also in a cancer context. The compound P3-25 was shown to be promising since able to inhibit the TRAF2-mediated NF-κB and AP-1 pathway activation [146]. Also, the silencing of copine 1 (CPNE1), a TRAF2 interactor, induced cell cycle arrest in PCa cells and this anti-tumor effect was reversed by overexpressing TRAF2 [147]. Tannic acid was able to downregulate several genes, including TRAF2, and to upregulate various pro-apoptotic genes, inhibiting PCa cell growth [148]. In rare cases, TRAF2 might be beneficial for cancers since it displays an anti-tumoral activity. A study showed that Sanggenon inhibits porcine reproductive and respiratory syndrome replication by suppressing NF-κB activation through increased TRAF2 expression [74]. Thereby, Sanggenon might be useful for mantle cell lymphoma, diffuse large B-cell lymphoma, multiple myeloma, some types of sarcomas and HCC associated with metabolic-associated fatty liver disease.

The anti-cancer therapeutic strategies targeting TRAF2, which either inhibit or activate its expression or target TRAF2 interactors, are outlined in Table 3.

**Table 3:** Anti-cancer therapeutic strategies targeting TRAF2 or TRAF2 interactors.

Therapeutic strategy	Effects on cancer cells and TME
TRAF2 silencing	•Induce growth arrest and reverse the radioresistant phenotype of glioblastoma cells [110] •Inhibits the migration and invasion of gastric cancer cells in vitro [121]
Copine 1 silencing (TRAF2 interactor)	•Induces cell cycle arrest in prostate cancer cells [142]
Anti-TNFR2 antibody BI-1808	• Depletes the TME of Tregs, modulates MDSCs and promotes the expansion of CD8 + T cells [128]
Anti-TNFR2 antibody APX602	• Reverses the immune suppressive characteristics of the TME [129]
Anti-Fn14 agonistic antibody	• Inhibits the growth of several cancer cell lines potentially through the depletion of TRAF2-cIAP1/2 [132]
Tetramethylpyrazine analog CXC195	• Induces apoptosis of bladder cancer cells [133]
Verproside	• Probably interacts with TRAF2/TRADD, inhibiting the activation of NF-kB in rhabdomyosarcoma cell lines [134]
TNIK inhibitor NCB-0846	• Inhibits TNIK and the EMT process in lung cancer and sensitizes TNIK-overexpressing lung squamous cell carcinoma to radiotherapy [136] • Hinders papillary thyroid carcinoma progression by hampering migration as a consequence of the inhibition of cytoskeletal remodeling [137]
TNIK inhibitor OBD9	• Induces autophagic degradation of TNIK and, subsequently, blocks the TCF4/β-catenin [139]
Wogonoside	• Downregulates TRAF2 and TRAF4 and, consequently, inactivates NF-kB and inhibits Twist expression and EMT [138]
Suramin analog NF023	• Disrupts the interaction between cIAP and TRAF2 and, consequently, inhibits NF-kB [14]
Liquidambaric acid (LDA)	• Targets TRAF2 and inhibits Wnt-β-catenin in colon cancer [40]
LDA analog	• Inhibits Wnt and TNF signaling and inhibits colon cancer cell growth in a mouse model [122]
Smac/DIABLO mimetic (cIAP1 and 2 inhibitors)	• Increases the efficacy of ICB in TRAF2-depleted cancer cells [125]
Xinfeng	• Downregulates TRAF2 and hampers NF-kB activation [140]
Putative NF-kB inhibitor P3-25	• Inhibits the TRAF2-mediated activation of NF-kB and AP-1 [141]
Tannic acid	• Downregulates several genes, including TRAF2, and upregulates various pro-apoptotic genes, inhibiting prostate cancer cell growth [143].
Sanggenon C	• Increases TRAF2 expression and NF-kB activation [74] • It might be useful for mantle cell lymphoma, diffuse large B-cell lymphoma, multiple myeloma, some types of sarcomas and HCC associated with metabolic-associated fatty liver disease
Peptidomimetics with the potential to inhibit the TRADD–TRAF2 interaction	• It might be useful in cancer treatment, but further studies are needed

## Future drug design

**Fig. 6:**
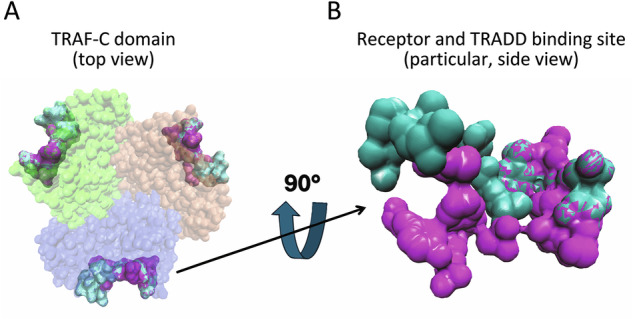
TRAF2-C surface structural features. **A** Schematic representation of trimeric TRAF2-C (top view) cartoon (PDB file 1ca4) with the three subunits in different colors (blue, green and orange). The surface residues involved in the interaction with TRADD or with CD40 receptor peptide are shown in cyan and in purple, respectively (obtained from PDB files 1f3v and 1qsc). **B** Details of the TRADD and/or CD40 binding residual surface (side view) obtained from panel (**A**) upon a rotation of 90°.

TRAF2 inhibition holds promise for reducing cancer aggressiveness, overcoming immune escape, and addressing resistance to conventional therapies, especially in combination with chemotherapy, radiotherapy, or immunotherapy. However, its biochemical and pharmacological properties remain poorly understood. Future drug design may benefit from exploring its structural and dynamic features. Comparative studies between TRAF1 and TRAF2 have revealed distinct affinities for proteins, such as TRADD, TANK and caspase 2, attributed to specific amino acids in their C-terminal domains. TRAF2’s uneven globular surface facilitates interactions with membrane receptors (e.g., TNFRs) and adaptor proteins (e.g., TRADD). Its rough outer surface offers anchoring points for medium-sized molecules, potentially enabling inhibition of its protein interactions (Fig. 6). Recent in silico studies have identified peptides capable of disrupting the TRAF2-TRADD interaction. Additionally, the dissociation of TRAF2’s trimeric structure into monomers under acidic conditions, such as binding to ganglioside-containing membranes (GM1) [145], is a process that is known to induce vesiculation [12, 13, 145]. This monomer-trimer equilibrium could serve as a therapeutic target, offering opportunities to modulate TRAF2 activity in vivo. These findings highlight TRAF2 as a promising yet underexplored target for future cancer therapy strategies.

## Conclusions

TRAF2 is a critical signaling hub involved in modulating pathways downstream of TNFR1 and TNFR2, influencing cell survival, apoptosis, inflammation, immune regulation, and cancer progression. TRAF2 supports macrophage activation, survival of DC, Treg and B cells, NK cell cytotoxicity, Th17 differentiation, and T-cell memory. Additionally, TRAF2 enhances the immunosuppressive functions of MDSCs in tumors, establishing its position as a key mediator of immune homeostasis and cancer immune evasion. In cancer, TRAF2 activates NF-κB to drive inflammation, angiogenesis, and immunosuppressive TME. It also supports tumor progression by promoting EMT and ER stress responses, enhancing resistance to apoptosis and anti-cancer therapies. Studying TRAF2 is crucial for understanding tumor signaling and interactions within the TME, including its role in immune dynamics, chemoresistance, and metastasis. Targeting TRAF2 remains challenging, but inhibitors, neutralizing antibodies, and peptides disrupting its interactions with TRADD or cIAPs show promise. Combining TRAF2 inhibition with chemotherapy or targeted therapies (VEGF, mTOR, or PD-L1 inhibitors) may enhance efficacy. These strategies offer the potential for tailored anti-cancer treatments that disrupt oncogenic pathways while preserving immune function. Further structural studies are needed to explore rare cases where TRAF2 may support anti-tumor immunity and to refine therapeutic approaches.

## References

[1] Noonan DM, De Lerma Barbaro A, Vannini N, Mortara L, Albini A. Inflammation, inflammatory cells and angiogenesis: decisions and indecisions. Cancer Metastasis Rev. 2008;27:31–40. 10.1007/s10555-007-9108-5. 18087678 10.1007/s10555-007-9108-5

[2] Darnay BG, Aggarwal BB. Signal transduction by tumour necrosis factor and tumour necrosis factor related ligands and their receptors. Ann Rheum Dis. 1999;58:I2–I13. 10.1136/ard.58.2008.i2. 10577967 10.1136/ard.58.2008.i2 PMC1766589

[3] Siegmund D, Wagner J, Wajant H. TNF receptor associated factor 2 (TRAF2) signaling in cancer. Cancers (Basel). 2022;14:4055. 10.3390/cancers14164055. 36011046 10.3390/cancers14164055 PMC9406534

[4] Park HH. Structure of TRAF Family: Current Understanding of Receptor Recognition. Front Immunol. 2018;9:1999. 10.3389/fimmu.2018.01999. 30214450 10.3389/fimmu.2018.01999 PMC6125299

[5] Speeckaert MM, Speeckaert R, Laute M, Vanholder R, Delanghe JR. Tumor necrosis factor receptors: biology and therapeutic potential in kidney diseases. Am J Nephrol. 2012;36:261–70. 10.1159/000342333. 22965073 10.1159/000342333

[6] Siegmund D, Lang I, Wajant H. Cell death-independent activities of the death receptors CD95, TRAILR1, and TRAILR2. FEBS J. 2017;284:1131–59. 10.1111/febs.13968. 27865080 10.1111/febs.13968

[7] Borghi A, Verstrepen L, Beyaert R. TRAF2 multitasking in TNF receptor-induced signaling to NF-κB, MAP kinases and cell death. Biochem Pharm. 2016;116:1–10. 10.1016/j.bcp.2016.03.009. 26993379 10.1016/j.bcp.2016.03.009

[8] Arthur JS, Ley SC. Mitogen-activated protein kinases in innate immunity. Nat Rev Immunol. 2013;13:679–92. 10.1038/nri3495. 23954936 10.1038/nri3495

[9] Fischer R, Kontermann RE, Pfizenmaier K. Selective targeting of TNF receptors as a novel therapeutic approach. Front Cell Dev Biol. 2020;8:401. 32528961 10.3389/fcell.2020.00401 PMC7264106

[10] Vince JE, Pantaki D, Feltham R, Mace PD, Cordier SM, Schmukle AC, et al. TRAF2 must bind to cellular inhibitors of apoptosis for tumor necrosis factor (tnf) to efficiently activate nf-{kappa}b and to prevent tnf-induced apoptosis. J Biol Chem. 2009;284:35906–15. 10.1074/jbc.M109.072256. 19815541 10.1074/jbc.M109.072256 PMC2791019

[11] Zhang R, Xu Y, Ekman N, Wu Z, Wu J, Alitalo K, et al. Etk/Bmx transactivates vascular endothelial growth factor 2 and recruits phosphatidylinositol 3-kinase to mediate the tumor necrosis factor-induced angiogenic pathway. J Biol Chem. 2003;278:51267–76. 10.1074/jbc.M310678200. 14532277 10.1074/jbc.M310678200

[12] Medler J, Wajant H. Tumor necrosis factor receptor-2 (TNFR2): An overview of an emerging drug target. Expert Opin Ther Targets. 2019;23:295–307. 10.1080/14728222.2019.1586886. 30856027 10.1080/14728222.2019.1586886

[13] Au PYB, Yeh WC. Physiological Roles and Mechanisms of Signaling by TRAF2 and TRAF5. In: Madame Curie Bioscience Database [Internet]. Austin (TX): Landes Bioscience; 2000-2013. Available from: https://www.ncbi.nlm.nih.gov/books/NBK6132/.

[14] Rothe M, Wong SC, Henzel WJ, Goeddel DV. A novel family of putative signal transducers associated with the cytoplasmic domain of the 75 kDa tumor necrosis factor receptor. Cell. 1994;78:681–92. 10.1016/0092-8674(94)90532-0. 8069916 10.1016/0092-8674(94)90532-0

[15] Di Venere A, Nicolai E, Minicozzi V, Caccuri AM, Di Paola L, Mei G. The Odd Faces of Oligomers: The Case of TRAF2-C, A Trimeric C-Terminal Domain of TNF Receptor-Associated Factor. Int J Mol Sci. 2021;22:5871. 10.3390/ijms22115871. 34070875 10.3390/ijms22115871 PMC8198530

[16] McWhirter SM, Pullen SS, Holton JM, Crute JJ, Kehry MR, Alber T. Crystallographic analysis of CD40 recognition and signaling by human TRAF2. Proc Natl Acad Sci USA. 1999;96:8408–13. 10.1073/pnas.96.15.8408. 10411888 10.1073/pnas.96.15.8408 PMC17529

[17] Zheng C, Kabaleeswaran V, Wang Y, Cheng G, Wu H. Crystal structures of the TRAF2: cIAP2 and the TRAF1: TRAF2: cIAP2 complexes: affinity, specificity, and regulation. Mol Cell. 2010;38:101–13. 10.1016/j.molcel.2010.03.009. 20385093 10.1016/j.molcel.2010.03.009 PMC2855162

[18] Minicozzi V, Di Venere A, Nicolai E, Giuliani A, Caccuri AM, Di Paola L, et al. Non-symmetrical structural behavior of a symmetric protein: the case of homo-trimeric TRAF2 (tumor necrosis factor-receptor associated factor 2). J Biomol Struct Dyn. 2021;39:319–29. 10.1080/07391102.2020.1719202. 31980009 10.1080/07391102.2020.1719202

[19] Ceccarelli A, Di Venere A, Nicolai E, De Luca A, Minicozzi V, Rosato N, et al. TNFR-Associated Factor-2 (TRAF2): Not Only a Trimer. Biochemistry. 2015;54:6153–61. 10.1021/acs.biochem.5b00674. 26390021 10.1021/acs.biochem.5b00674

[20] Erba F, Di Paola L, Di Venere A, Mastrangelo E, Cossu F, Mei G, et al. Head or tail? A molecular dynamics approach to the complex structure of TNF-associated factor TRAF2. Biomol Concepts. 2023;14. 10.1515/bmc-2022-0031. 10.1515/bmc-2022-0031 37377424

[21] Ceccarelli A, Di Venere A, Nicolai E, De Luca A, Rosato N, Gratton E, et al. New insight into the interaction of TRAF2 C-terminal domain with lipid raft microdomains. Biochim Biophys Acta Mol Cell Biol Lipids. 2017;1862:813–22. 10.1016/j.bbalip.2017.05.003. 28499815 10.1016/j.bbalip.2017.05.003 PMC5562040

[22] Di Venere A, Nicolai E, Sinibaldi F, Di Pierro D, Caccuri AM, Mei G. Studying the TRAF2 binding to model membranes: The role of subunits dissociation. Biotechnol Appl Biochem. 2018;65:38–45. 10.1002/bab.1615. 28960521 10.1002/bab.1615

[23] Cossu F, Sorrentino L, Fagnani E, Zaffaroni M, Milani M, Giorgino T, et al. Computational and experimental characterization of NF023, a candidate anticancer compound inhibiting cIAP2/TRAF2 assembly. J Chem Inf Model. 2020;60:5036–44. 10.1021/acs.jcim.0c00518. 32820924 10.1021/acs.jcim.0c00518

[24] Shi JH, Sun SC. Tumor necrosis factor receptor-associated factor regulation of nuclear factor κB and mitogen-activated protein kinase pathways. Front Immunol. 2018;9:1849. 10.3389/fimmu.2018.01849. 30140268 10.3389/fimmu.2018.01849 PMC6094638

[25] Grech AP, Amesbury M, Chan T, Gardam S, Basten A, Brink R. TRAF2 differentially regulates the canonical and noncanonical pathways of NF-kappaB activation in mature B cells. Immunity. 2004;21:629–42. 10.1016/j.immuni.2004.09.011. 15539150 10.1016/j.immuni.2004.09.011

[26] Li S, Wang D, Zhao J, Weathington NM, Shang D, Zhao Y. The deubiquitinating enzyme USP48 stabilizes TRAF2 and reduces E-cadherin-mediated adherens junctions. FASEB J. 2018;32:230–42. 10.1096/fj.201700415RR. 28874458 10.1096/fj.201700415RR PMC5731130

[27] Xu D, Zhao H, Jin M, Zhu H, Shan B, Geng J, et al. Modulating TRADD to restore cellular homeostasis and inhibit apoptosis. Nature. 2020;587:133–8. 10.1038/s41586-020-2757-z. 32968279 10.1038/s41586-020-2757-z

[28] Junjappa RP, Patil P, Bhattarai KR, Kim HR, Chae HJ. IRE1α implications in endoplasmic reticulum stress-mediated development and pathogenesis of autoimmune diseases. Front Immunol. 2018;9:1289. 10.3389/fimmu.2018.01289. 29928282 10.3389/fimmu.2018.01289 PMC5997832

[29] Yang KC, Ma X, Liu H, Murphy J, Barger PM, Mann DL, et al. Tumor necrosis factor receptor-associated factor 2 mediates mitochondrial autophagy. Circ Heart Fail. 2015;8:175–87. 10.1161/CIRCHEARTFAILURE.114.001635. 25339503 10.1161/CIRCHEARTFAILURE.114.001635 PMC4303508

[30] Dhillon B, Aleithan F, Abdul-Sater Z, Abdul-Sater AA. The evolving role of TRAFs in mediating inflammatory responses. Front Immunol. 2019;10:104. 10.3389/fimmu.2019.00104. 30778351 10.3389/fimmu.2019.00104 PMC6369152

[31] Alam MS, Otsuka S, Wong N, Abbasi A, Gaida MM, Fan Y, et al. TNF plays a crucial role in inflammation by signaling via T cell TNFR2. Proc Natl Acad Sci USA. 2021;118:e2109972118. 10.1073/pnas.2109972118. 34873037 10.1073/pnas.2109972118 PMC8685675

[32] Yang XD, Sun SC. Targeting signaling factors for degradation, an emerging mechanism for TRAF functions. Immunol Rev. 2015;266:56–71. 10.1111/imr.12311. 26085207 10.1111/imr.12311 PMC4473799

[33] Habelhah H, Takahashi S, Cho SG, Kadoya T, Watanabe T, Ronai Z. Ubiquitination and translocation of TRAF2 is required for activation of JNK but not of p38 or NF-kappaB. EMBO J. 2004;23:322–32. 10.1038/sj.emboj.7600044. 14713952 10.1038/sj.emboj.7600044 PMC1271753

[34] Wajant H, Siegmund D. TNFR1 and TNFR2 in the control of the life and death balance of macrophages. Front Cell Dev Biol. 2019;7:91. 10.3389/fcell.2019.00091. 31192209 10.3389/fcell.2019.00091 PMC6548990

[35] Yamamoto S, Iwakuma T. RIPK1-TRAF2 interplay on the TNF/NF-κB signaling, cell death, and cancer development in the liver. Transl Cancer Res. 2017;6:94–109. 10.21037/tcr.2017.04.01. 30123738 10.21037/tcr.2017.04.01 PMC6097634

[36] Tang Y, Tu H, Zhang J, Zhao X, Wang Y, Qin J, et al. K63-linked ubiquitination regulates RIPK1 kinase activity to prevent cell death during embryogenesis and inflammation. Nat Commun. 2019;10:4157. 10.1038/s41467-019-12033-8. 31519887 10.1038/s41467-019-12033-8 PMC6744441

[37] Mihaly SR, Ninomiya-Tsuji J, Morioka S. TAK1 control of cell death. Cell Death Differ. 2014;21:1667–76. 10.1038/cdd.2014.123. 25146924 10.1038/cdd.2014.123 PMC4211365

[38] Sabio G, Davis RJ. TNF and MAP kinase signalling pathways. Semin Immunol. 2014;26:237–45. 10.1016/j.smim.2014.02.009. 24647229 10.1016/j.smim.2014.02.009 PMC4099309

[39] Fan Y, Yu Y, Shi Y, Sun W, Xie M, Ge N, et al. Lysine 63-linked polyubiquitination of TAK1 at lysine 158 is required for tumor necrosis factor alpha- and interleukin-1beta-induced IKK/NF-kappaB and JNK/AP-1 activation. J Biol Chem. 2010;285:5347–60. 10.1074/jbc.M109.076976. 20038579 10.1074/jbc.M109.076976 PMC2820763

[40] Zelova H, Hosek J. TNF-alpha signalling and inflammation: interactions between old acquaintances. Inflamm Res. 2013;62:641–51. 23685857 10.1007/s00011-013-0633-0

[41] Takaesu G, Kishida S, Hiyama A, Yamaguchi K, Shibuya H, Irie K, et al. TAB2, a novel adaptor protein, mediates activation of TAK1 MAPKKK by linking TAK1 to TRAF6 in the IL-1 signal transduction pathway. Mol Cell. 2000;5:649–58. 10.1016/s1097-2765(00)80244-0. 10882101 10.1016/s1097-2765(00)80244-0

[42] Sun SC. Controlling the fate of NIK: a central stage in noncanonical NF-kappaB signaling. Sci Signal. 2010;3:pe18. 10.1126/scisignal.3123pe18. 20501935 10.1126/scisignal.3123pe18 PMC5753754

[43] Jiang Y, Yu M, Hu X, Han L, Yang K, Ba H, et al. STAT1 mediates transmembrane TNF-alpha-induced formation of death-inducing signaling complex and apoptotic signaling via TNFR1. Cell Death Differ. 2017;24:660–71. 10.1038/cdd.2016.162. 28186502 10.1038/cdd.2016.162 PMC5384023

[44] Gonzalvez F, Lawrence D, Yang B, Yee S, Pitti R, Marsters S, et al. TRAF2 Sets a threshold for extrinsic apoptosis by tagging caspase-8 with a ubiquitin shutoff timer. Mol Cell. 2012;48:888–99. 23142077 10.1016/j.molcel.2012.09.031

[45] Sun L, Wang H, Wang Z, He S, Chen S, Liao D, et al. Mixed lineage kinase domain-like protein mediates necrosis signaling downstream of RIP3 kinase. Cell. 2012;148:213–27. 10.1016/j.cell.2011.11.031. 22265413 10.1016/j.cell.2011.11.031

[46] Ye LL, Wei XS, Zhang M, Niu YR, Zhou Q. The significance of tumor necrosis factor receptor type II in CD8+ regulatory T cells and CD8+ effector T cells. Front Immunol. 2018;9:583. 10.3389/fimmu.2018.00583. 29623079 10.3389/fimmu.2018.00583 PMC5874323

[47] Festjens N, Vanden Berghe T, Vandenabeele P. Necrosis, a well-orchestrated form of cell demise: Signalling cascades, important mediators and concomitant immune response. Biochim Biophys Acta. 2006;1757:1371–87. 16950166 10.1016/j.bbabio.2006.06.014

[48] Wang L, Chang X, Feng J, Yu J, Chen G. TRADD mediates RIPK1-independent necroptosis induced by tumor necrosis factor. Front Cell Dev Biol. 2020;7:393. 10.3389/fcell.2019.00393. 32039207 10.3389/fcell.2019.00393 PMC6987388

[49] Pobezinskaya YL, Liu Z. The role of TRADD in death receptor signaling. Cell Cycle. 2012;11:871–6. 10.4161/cc.11.5.19300. 22333735 10.4161/cc.11.5.19300 PMC3679287

[50] Li Z, Yuan W, Lin Z. Functional roles in cell signaling of adaptor protein TRADD from a structural perspective. Comput Struct Biotechnol J. 2020;16:2867–76. 10.1016/j.csbj.2020.10.008. 10.1016/j.csbj.2020.10.008 PMC7593343 33163147

[51] Hernández AR, Klein AM, Kirschner MW. Kinetic responses of β-catenin specify the sites of Wnt control. Science. 2012;338:1337–40. 10.1126/science.1228734. 23138978 10.1126/science.1228734

[52] Mahmoudi T, Li VSW, Ng SS, Taouatas N, Vries RGJ, Mohammed S, et al. The kinase TNIK is an essential activator of Wnt target genes. EMBO J. 2009;28:3329–40. 10.1038/emboj.2009.285. 19816403 10.1038/emboj.2009.285 PMC2776109

[53] Yan R, Zhu H, Huang P, Yang M, Shen M, Pan Y, et al. Liquidambaric acid inhibits Wnt/β-catenin signaling and colon cancer via targeting TNF receptor-associated factor 2. Cell Rep. 2022;38:110319. 10.1016/j.celrep.2022.110319. 35108540 10.1016/j.celrep.2022.110319

[54] Yoon M, Kim E, Seo SH, Kim G-U, Choi K-Y. KY19382 accelerates cutaneous wound healing via activation of the Wnt/β-catenin signaling pathway. Int Mol Sci. 2023;24:11742. 10.3390/ijms241411742. 10.3390/ijms241411742 PMC10380997 37511501

[55] van Amerongen R, Fuerer C, Mizutani M, Nusse R. Wnt5a can both activate and repress Wnt/β-catenin signaling during mouse embryonic development. Dev Biol. 2012;369:101–14. 10.1016/j.ydbio.2012.06.020. 22771246 10.1016/j.ydbio.2012.06.020 PMC3435145

[56] Konopelski Snavely SE, Srinivasan S, Dreyer CA, Tan J, Carraway KL 3rd, Ho HH. Non-canonical WNT5A-ROR signaling: New perspectives on an ancient developmental pathway. Curr Top Dev Biol. 2023;153:195–227. 10.1016/bs.ctdb.2023.01.009. 36967195 10.1016/bs.ctdb.2023.01.009 PMC11042798

[57] Barbero G, Castro MV, Villanueva MB, Quezada MJ, Fernández NB, DeMorrow S, et al. An autocrine Wnt5a loop promotes NF-κB pathway activation and cytokine/chemokine secretion in melanoma. Cells. 2019;8:1060. 10.3390/cells8091060. 31510045 10.3390/cells8091060 PMC6770184

[58] Schmitz ML, Shaban MS, Albert BV, Gökçen A, Kracht M. The crosstalk of endoplasmic reticulum (ER) stress pathways with NF-κB: complex mechanisms relevant for cancer, inflammation and infection. Biomedicines. 2018;6:58. 10.3390/biomedicines6020058. 29772680 10.3390/biomedicines6020058 PMC6027367

[59] Tong Q, Wu L, Jiang T, Ou Z, Zhang Y, Zhu D. Inhibition of endoplasmic reticulum stress-activated IRE1α-TRAF2-caspase-12 apoptotic pathway is involved in the neuroprotective effects of telmisartan in the rotenone rat model of Parkinson’s disease. Eur J Pharm. 2016;776:106–15. 10.1016/j.ejphar.2016.02.042. 10.1016/j.ejphar.2016.02.042 26879867

[60] Ruder B, Atreya R, Becker C. Tumour necrosis factor alpha in intestinal homeostasis and gut related diseases. Int J Mol Sci. 2019;20:1887. 10.3390/ijms20081887. 30995806 10.3390/ijms20081887 PMC6515381

[61] De Luca A, Mei G, Rosato N, Nicolai E, Federici L, Palumbo C, et al. The fine-tuning of TRAF2-GSTP1-1 interaction: effect of ligand binding and in situ detection of the complex. Cell Death Dis. 2014;5:e1015. 10.1038/cddis.2013.529. 24457959 10.1038/cddis.2013.529 PMC4040697

[62] Dupoux A, Cartier J, Cathelin S, Filomenko R, Solary E, Dubrez-Daloz L. cIAP1-dependent TRAF2 degradation regulates the differentiation of monocytes into macrophages and their response to CD40 ligand. Blood. 2009;113:175–85. 10.1182/blood-2008-02-137919. 18827186 10.1182/blood-2008-02-137919 PMC2951832

[63] Xie X, Jin J, Zhu L, Jie Z, Li Y, Zhao B, et al. Cell type-specific function of TRAF2 and TRAF3 in regulating type I IFN induction. Cell Biosci. 2019;9:5. 10.1186/s13578-018-0268-5. 30622699 10.1186/s13578-018-0268-5 PMC6318904

[64] Gandolfi S, Dufva O, Huuhtanen J, Dashevsky O, Klievink J, Bouhlal J, et al. Functional genomic landscape of natural killer cell evasion in multiple myeloma. Blood. 2021;138:732. 10.1182/blood-2021-153925.

[65] Haxhinasto S, Mathis D, Benoist C. The AKT-mTOR axis regulates de novo differentiation of CD4+Foxp3+ cells. J Exp Med. 2008;205:565–74. 10.1084/jem.20071477. 18283119 10.1084/jem.20071477 PMC2275380

[66] Miller PG, Bonn MB, McKarns SC. Transmembrane TNF-TNFR2 impairs Th17 differentiation by promoting Il2 expression. J Immunol. 2015;195:2633–47. 26268655 10.4049/jimmunol.1500286 PMC4841279

[67] Yan L, Zheng D, Xu RH. Critical role of tumor necrosis factor signaling in mesenchymal stem cell-based therapy for autoimmune and inflammatory diseases. Front Immunol. 2018;9:1658. 10.3389/fimmu.2018.01658. 30079066 10.3389/fimmu.2018.01658 PMC6062591

[68] Bishop GA. The multifaceted roles of TRAFs in the regulation of B-cell function. Nat Rev Immunol. 2004;4:775–86. 10.1038/nri1462. 15459669 10.1038/nri1462

[69] Merluzzi S, D’Orlando O, Leonardi A, Vitale G, Pucillo C. TRAF2 and p38 are involved in B cells CD40-mediated APE/Ref-1 nuclear translocation: a novel pathway in B cell activation. Mol Immunol. 2008;45:76–86. 10.1016/j.molimm.2007.05.010. 17599408 10.1016/j.molimm.2007.05.010

[70] Harit K, Bhattacharjee R, Matuschewski K, Becker J, Kalinke U, Schlüter D, et al. The deubiquitinating enzyme OTUD7b protects dendritic cells from TNF-induced apoptosis by stabilizing the E3 ligase TRAF2. Cell Death Dis. 2023;14:480. 10.1038/s41419-023-06014-5. 37516734 10.1038/s41419-023-06014-5 PMC10387084

[71] Jin J, Xiao Y, Hu H, Zou Q, Li Y, Gao Y, et al. Proinflammatory TLR signalling is regulated by a TRAF2-dependent proteolysis mechanism in macrophages. Nat Commun. 2015;6:5930. 10.1038/ncomms6930. 25565375 10.1038/ncomms6930 PMC4286812

[72] Lu CH, Yeh DW, Lai CY, Liu YL, Huang LR, Lee AY, et al. USP17 mediates macrophage-promoted inflammation and stemness in lung cancer cells by regulating TRAF2/TRAF3 complex formation. Oncogene. 2018;37:6327–40. 10.1038/s41388-018-0411-0. 30038267 10.1038/s41388-018-0411-0 PMC6283856

[73] Polz J, Remke A, Weber S, Schmidt D, Weber-Steffens D, Pietryga-Krieger A, et al. Myeloid suppressor cells require membrane TNFR2 expression for suppressive activity. Immun Inflamm Dis. 2014;2:121–30. 10.1002/iid3.19. 25400932 10.1002/iid3.19 PMC4217546

[74] Liu X, Zhu Y, Wang D, Feng R, Chen Z, Zheng Z, et al. The natural compound Sanggenon C inhibits PRRSV infection by regulating the TRAF2/NF-κB signalling pathway. Vet Res. 2023;54:114. 10.1186/s13567-023-01245-y. 38037100 10.1186/s13567-023-01245-y PMC10691163

[75] Xie X, Zhu L, Jie Z, Li Y, Gu M, Zhou X, et al. TRAF2 regulates T cell immunity by maintaining a Tpl2-ERK survival signaling axis in effector and memory CD8 T cells. Cell Mol Immunol. 2021;18:2262–74. 10.1038/s41423-020-00583-7. 33203937 10.1038/s41423-020-00583-7 PMC8429472

[76] Shen RR, Zhou AY, Kim E, O’Connell JT, Hagerstrand D, Beroukhim R, et al. TRAF2 is an NF-κB-activating oncogene in epithelial cancers. Oncogene. 2015;34:209–16. 24362534 10.1038/onc.2013.543 PMC4067463

[77] Zhang W, Sun Y, Liu L, Li Z. Prognostic significance of TNFR-associated factor 1 and 2 (TRAF1 and TRAF2) in glioblastoma. Med Sci Monit. 2017;23:4506–12. 10.12659/msm.903397. 28926524 10.12659/MSM.903397 PMC5616136

[78] Wei B, Ruan J, Mi Y, Hu J, Zhang J, Wang Z, et al. Knockdown of TNF receptor-associated factor 2 (TRAF2) modulates in vitro growth of TRAIL-treated prostate cancer cells. Biomed Pharmacother. 2017;93:462–9. 10.1016/j.biopha.2017.05.145. 28667915 10.1016/j.biopha.2017.05.145

[79] Compagno M, Lim WK, Grunn A, Nandula SV, Brahmachary M, Shen Q, et al. Mutations of multiple genes cause deregulation of NF-kappaB in diffuse large B-cell lymphoma. Nature. 2009;459:717–21. 10.1038/nature07968. 19412164 10.1038/nature07968 PMC2973325

[80] Keats JJ, Fonseca R, Chesi M, Schop R, Baker A, Chng WJ, et al. Promiscuous mutations activate the noncanonical NF-kappaB pathway in multiple myeloma. Cancer Cell. 2007;12:131–44. 10.1016/j.ccr.2007.07.003. 17692805 10.1016/j.ccr.2007.07.003 PMC2083698

[81] Takahashi H, Yoshimatsu G, Faustman DL. The roles of TNFR2 signaling in cancer cells and the tumor microenvironment and the potency of TNFR2 targeted therapy. Cells. 2022;11:1952. 10.3390/cells11121952. 35741080 10.3390/cells11121952 PMC9222015

[82] Deen AJ, Adinolfi S, Härkönen J, Patinen T, Liu X, Laitinen T, et al. Oncogenic KEAP1 mutations activate TRAF2-NFκB signaling to prevent apoptosis in lung cancer cells. Redox Biol. 2024;69:103031. 10.1016/j.redox.2024.103031. 38184997 10.1016/j.redox.2024.103031 PMC10808971

[83] Xu Y, Li L, Yang W, Zhang K, Zhang Z, Yu C, et al. TRAF2 promotes M2-polarized tumor-associated macrophage infiltration, angiogenesis and cancer progression by inhibiting autophagy in clear cell renal cell carcinoma. J Exp Clin Cancer Res. 2023;42:159. 10.1186/s13046-023-02742-w. 37415241 10.1186/s13046-023-02742-w PMC10324183

[84] Choi JM, Devkota S, Sung YH, Lee HW. EI24 regulates epithelial-to-mesenchymal transition and tumor progression by suppressing TRAF2-mediated NF-κB activity. Oncotarget. 2013;4:2383–96. 10.18632/oncotarget.1434. 24280371 10.18632/oncotarget.1434 PMC3926834

[85] Zhang T, Ma C, Zhang Z, Zhang H, Hu H. NF-κB signaling in inflammation and cancer. MedComm. 2021;2:618–53. 10.1002/mco2.104. 34977871 10.1002/mco2.104 PMC8706767

[86] Greten FR, Grivennikov SI. Inflammation and cancer: triggers, mechanisms, and consequences. Immunity. 2019;51:27–41. 10.1016/j.immuni.2019.06.025. 31315034 10.1016/j.immuni.2019.06.025 PMC6831096

[87] Albini A. Tumor microenvironment, a dangerous society leading to cancer metastasis. From mechanisms to therapy and prevention. Cancer Metastasis Rev. 2008;27:3–4. 10.1007/s10555-007-9102-y. 18043872 10.1007/s10555-007-9102-y

[88] Li L, Yu R, Cai T, Chen Z, Lan M, Zou T, et al. Effects of immune cells and cytokines on inflammation and immunosuppression in the tumor microenvironment. Int Immunopharmacol. 2020;88:106939. 10.1016/j.intimp.2020.106939. 33182039 10.1016/j.intimp.2020.106939

[89] Pires BR, Mencalha AL, Ferreira GM, de Souza WF, Morgado-Díaz JA, Maia AM, et al. NF-kappaB is involved in the regulation of EMT genes in breast cancer cells. PLoS One. 2017;12:e0169622. 10.1371/journal.pone.0169622. 28107418 10.1371/journal.pone.0169622 PMC5249109

[90] Ribatti D, Tamma R, Annese T. Epithelial-mesenchymal transition in cancer: a historical overview. Transl Oncol. 2020;13:100773. 10.1016/j.tranon.2020.100773. 32334405 10.1016/j.tranon.2020.100773 PMC7182759

[91] Zhang TT, Yi W, Dong DZ, Ren ZY, Zhang Y, Du F. METTL3-mediated upregulation of FAM135B promotes EMT of esophageal squamous cell carcinoma via regulating the Wnt/β-catenin pathway. Am J Physiol Cell Physiol. 2024;327:C329–C340. 10.1152/ajpcell.00529.2023. 38881420 10.1152/ajpcell.00529.2023

[92] Corso G. Pleiotropic cancer manifestations of germline CDH1 mutations: risks and management. J Surg Oncol. 2022;125:1326–31. 10.1002/jso.26847. 35277969 10.1002/jso.26847 PMC9313879

[93] Corso G, Corso F, Bellerba F, Carneiro P, Seixas S, Cioffi A, et al. Geographical distribution of E-cadherin germline mutations in the context of diffuse gastric cancer: a systematic review. Cancers (Basel). 2021;13:1269. 10.3390/cancers13061269. 33809393 10.3390/cancers13061269 PMC8001745

[94] Corso G, Carvalho J, Marrelli D, Vindigni C, Carvalho B, Seruca R, et al. Somatic mutations and deletions of the E-cadherin gene predict poor survival of patients with gastric cancer. J Clin Oncol. 2013;31:868–75. 10.1200/JCO.2012.44.4612. 23341533 10.1200/JCO.2012.44.4612

[95] Abaurrea A, Araujo AM, Caffarel MM. The role of the IL-6 cytokine family in epithelial-mesenchymal plasticity in cancer progression. Int J Mol Sci. 2021;22:8334. 10.3390/ijms22158334. 34361105 10.3390/ijms22158334 PMC8347315

[96] Chen X, Cubillos-Ruiz JR. Endoplasmic reticulum stress signals in the tumour and its microenvironment. Nat Rev Cancer. 2021;21:71–88. 10.1038/s41568-020-00312-2. 33214692 10.1038/s41568-020-00312-2 PMC7927882

[97] Sano R, Reed JC. ER stress-induced cell death mechanisms. Biochim Biophys Acta. 2013;1833:3460–70. 10.1016/j.bbamcr.2013.06.028. 23850759 10.1016/j.bbamcr.2013.06.028 PMC3834229

[98] Zhang W, Shi Y, Oyang L, Cui S, Li S, Li J, et al. Endoplasmic reticulum stress-a key guardian in cancer. Cell Death Discov. 2024;10:343. 10.1038/s41420-024-02110-3. 39080273 10.1038/s41420-024-02110-3 PMC11289465

[99] Verfaillie T, Salazar M, Velasco G, Agostinis P. Linking ER stress to autophagy: potential implications for cancer therapy. Int J Cell Biol. 2010;2010:930509. 10.1155/2010/930509. 20145727 10.1155/2010/930509 PMC2817393

[100] Liu JY, Zeng QH, Cao PG, Xie D, Chen X, Yang F, et al. RIPK4 promotes bladder urothelial carcinoma cell aggressiveness by upregulating VEGF-A through the NF-κB pathway. Br J Cancer. 2018;118:1617–27. 10.1038/s41416-018-0116-8. 29867225 10.1038/s41416-018-0116-8 PMC6008479

[101] Bourhis M, Palle J, Galy-Fauroux I, Terme M. Direct and indirect modulation of T Cells by VEGF-A counteracted by anti-angiogenic treatment. Front Immunol. 2021;12:616837. 10.3389/fimmu.2021.616837. 33854498 10.3389/fimmu.2021.616837 PMC8039365

[102] Zhao X, Rong L, Zhao X, Li X, Liu X, Deng J, et al. TNF signaling drives myeloid-derived suppressor cell accumulation. J Clin Invest. 2012;122:4094–104. 23064360 10.1172/JCI64115 PMC3484453

[103] Zou W, Restifo NP. TH 17 cells in tumour immunity and immunotherapy. Nat Rev Immunol. 2010;10:248–56. 20336152 10.1038/nri2742 PMC3242804

[104] Villanueva JE, Walters SN, Saito M, Malle EK, Zammit NW, Watson KA, et al. Targeted deletion of Traf2 allows immunosuppression-free islet allograft survival in mice. Diabetologia. 2017;60:679–89. 10.1007/s00125-016-4198-7. 28062921 10.1007/s00125-016-4198-7

[105] Guéry L, Hugues S. Th17 cell plasticity and functions in cancer immunity. BioMed Res Int. 2015;2015:1. 10.1155/2015/314620 PMC4637016 26583099

[106] Zhu H, Xuan Y, Huang P, Zhang C, Ke X, Qu Y, et al. Liquidambaric lactone is a potent inhibitor of TRAF2 for cancer therapy. Pharm Res Mod Chin Med. 2023;7:100265. 10.1016/j.prmcm.2023.100265.

[107] Zhou N, Guo C, Li X, Tu L, Du J, Qian Q, et al. USP24 promotes hepatocellular carcinoma tumorigenesis through deubiquitinating and stabilizing TRAF2. Biochem Pharm. 2024;229:116473. 10.1016/j.bcp.2024.116473. 39127151 10.1016/j.bcp.2024.116473

[108] Wang Z, Zhang Y, Shen Y, Zhou H, Gao Y, Zhu C, et al. Unlocking hepatocellular carcinoma aggression: STAMBPL1-mediated TRAF2 deubiquitination activates WNT/PI3K/NF-kb signaling pathway. Biol Direct. 2024;19:18. 10.1186/s13062-024-00460-7. 38419066 10.1186/s13062-024-00460-7 PMC10903047

[109] Yao J, Liang X, Xu S, Liu Y, Shui L, Li S, et al. TRAF2 inhibits senescence in hepatocellular carcinoma cells via regulating the ROMO1/ NAD+/SIRT3/SOD2 axis. Free Radic Biol Med. 2024;211:47–62. 10.1016/j.freeradbiomed.2023.11.035. 38043870 10.1016/j.freeradbiomed.2023.11.035

[110] Liang X, Yao J, Cui D, Zheng W, Liu Y, Lou G, et al. The TRAF2-p62 axis promotes proliferation and survival of liver cancer by activating mTORC1 pathway. Cell Death Differ. 2023;30:1550–62. 10.1038/s41418-023-01164-7. 37081115 10.1038/s41418-023-01164-7 PMC10244464

[111] Khaled J, Kopsida M, Lennernäs H, Heindryckx F. Drug resistance and endoplasmic reticulum stress in hepatocellular carcinoma. Cells. 2022;11:632. 10.3390/cells11040632. 35203283 10.3390/cells11040632 PMC8870354

[112] Tao L, Ren X, Zhai W, Chen Z. Progress and prospects of noncanonical NF-κB signaling pathway in the regulation of liver diseases. Molecules. 2022;27:4275. 10.3390/molecules27134275. 35807520 10.3390/molecules27134275 PMC9268066

[113] Cho HA, Park IS, Kim TW, Oh YK, Yang KS, Kim JS. Suppression of hepatitis B virus-derived human hepatocellular carcinoma by NF-kappaB-inducing kinase-specific siRNA using liver-targeting liposomes. Arch Pharm Res. 2009;32:1077–86. 10.1007/s12272-009-1714-z. 19641890 10.1007/s12272-009-1714-z

[114] Tan HY, Wang N, Man K, Tsao SW, Che CM, Feng Y. Autophagy-induced RelB/p52 activation mediates tumour-associated macrophage repolarisation and suppression of hepatocellular carcinoma by natural compound baicalin. Cell Death Dis. 2015;6:e1942. 10.1038/cddis.2015.271. 26492375 10.1038/cddis.2015.271 PMC4632300

[115] Zhang YS, Chen T, Cai YJ, Dong J, Bai F, Gao X, et al. MicroRNA-647 promotes the therapeutic effectiveness of argon-helium cryoablation and inhibits cell proliferation through targeting TRAF2 via the NF-κB signaling pathway in non-small cell lung cancer. Onco Targets Ther. 2018;11:6777–84. 10.2147/OTT.S159337. 30349310 10.2147/OTT.S159337 PMC6188019

[116] Zheng M, Morgan-Lappe SE, Yang J, Bockbrader KM, Pamarthy D, Thomas D, et al. Growth inhibition and radiosensitization of glioblastoma and lung cancer cells by small interfering RNA silencing of tumor necrosis factor receptor-associated factor 2. Cancer Res. 2008;68:7570–8. 10.1158/0008-5472.CAN-08-0632. 18794145 10.1158/0008-5472.CAN-08-0632 PMC2597026

[117] Zhang MH, Zhang HH, Du XH, Gao J, Li C, Shi HR, et al. UCHL3 promotes ovarian cancer progression by stabilizing TRAF2 to activate the NF-κB pathway. Oncogene. 2020;39:322–33. 10.1038/s41388-019-0987-z. 31477831 10.1038/s41388-019-0987-z

[118] Zhu H, Ding W, Wu J, Ma R, Pan Z, Mao X. TRAF2 knockdown in nasopharyngeal carcinoma induced cell cycle arrest and enhanced the sensitivity to radiotherapy. Biomed Res Int. 2020;2020:1641340. 10.1155/2020/1641340. 32566659 10.1155/2020/1641340 PMC7277071

[119] Tam SY, Wu VWC, Law HKW. JNK pathway mediates low oxygen level-induced epithelial-mesenchymal transition and stemness maintenance in colorectal cancer cells. Cancers. 2020;12:224. 10.3390/cancers12010224. 31963305 10.3390/cancers12010224 PMC7017419

[120] Peramuhendige P, Marino S, Bishop RT, de Ridder D, Khogeer A, Baldini I, et al. TRAF2 in osteotropic breast cancer cells enhances skeletal tumour growth and promotes osteolysis. Sci Rep. 2018;8:39. 29311633 10.1038/s41598-017-18327-5 PMC5758572

[121] da Silva SD, Xu B, Maschietto M, Marchi FA, Alkailani MI, Bijian K, et al. TRAF2 cooperates with focal adhesion signaling to regulate cancer cell susceptibility to anoikis. Mol Cancer Ther. 2019;18:139–46. 10.1158/1535-7163.MCT-17-1261. 30373932 10.1158/1535-7163.MCT-17-1261

[122] Karpisheh V, Ahmadi M, Abbaszadeh-Goudarzi K, Mohammadpour Saray M, Barshidi A, Mohammadi H, et al. The role of Th17 cells in the pathogenesis and treatment of breast cancer. Cancer Cell Int. 2022;22:108. 10.1186/s12935-022-02528-8. 35248028 10.1186/s12935-022-02528-8 PMC8897940

[123] Gu Y, Liang C. TRAIP suppressed apoptosis and cell cycle to promote prostate cancer proliferation via TRAF2-PI3K-AKT pathway activation. Int Urol Nephrol. 2024;56:1639–48. 10.1007/s11255-023-03890-w. 38100027 10.1007/s11255-023-03890-w

[124] Du J, Du Y, Chen L, Liu H. IL-17 promotes melanoma through TRAF2 as a scaffold protein recruiting PIAS2 and ELAVL1 to induce EPHA5. Biochim Biophys Acta Mol Cell Res. 2023;1870:119547. 10.1016/j.bbamcr.2023.119547. 37481078 10.1016/j.bbamcr.2023.119547

[125] Vashisht M, Ge H, John J, McKelvey HA, Chen J, Chen Z, et al. TRAF2/3 deficient B cells resist DNA damage-induced apoptosis via NF-κB2/XIAP/cIAP2 axis and IAP antagonist sensitizes mutant lymphomas to chemotherapeutic drugs. Cell Death Dis. 2023;14:599. 10.1038/s41419-023-06122-2. 37679334 10.1038/s41419-023-06122-2 PMC10485046

[126] Schürch C, Riether C, Matter MS, Tzankov A, Ochsenbein AF. CD27 signaling on chronic myelogenous leukemia stem cells activates Wnt target genes and promotes disease progression. J Clin Invest. 2012;122:624–38. 10.1172/JCI45977. 22232214 10.1172/JCI45977 PMC3266773

[127] Martinez-Forero I, Azpilikueta A, Bolaños-Mateo E, Nistal-Villan E, Palazon A, Teijeira A, et al. T cell costimulation with anti-CD137 monoclonal antibodies is mediated by K63-polyubiquitin-dependent signals from endosomes. J Immunol. 2013;190:6694–706. 23690480 10.4049/jimmunol.1203010

[128] Vredevoogd DW, Kuilman T, Ligtenberg MA, Boshuizen J, Stecker KE, de Bruijn B, et al. Augmenting immunotherapy impact by lowering tumor TNF cytotoxicity threshold. Cell. 2019;178:585–599.e15. 10.1016/j.cell.2019.06.014. 31303383 10.1016/j.cell.2019.06.014

[129] Zhao XY, Wang XY, Wei QY, Xu YM, Lau ATY. Potency and selectivity of SMAC/DIABLO mimetics in solid tumor therapy. Cells. 2020;9:1012. 10.3390/cells9041012. 32325691 10.3390/cells9041012 PMC7226512

[130] Mårtensson L, Kovacek M, Holmkvist P, Semmrich M, Svensson C, Blidberg T, et al. Pre-clinical development of TNFR2 ligand-blocking BI-1808 for cancer immunotherapy. J Immunother Cancer 2020;8. 10.1136/jitc-2020-SITC2020.0725.

[131] Krishnan S, Alvarado R, Huang G, Yang X, Filbert EL. APX601, a potent TNFR2 antagonist as a novel and promising approach to reverse tumor immune suppression. Cancer Res. 2021;81:LB175. 10.1158/1538-7445.AM2021-LB175.

[132] Debnath S, Sarkar A, Mukherjee DD, Ray S, Mahata B, Mahata T, et al. Eriodictyol mediated selective targeting of the TNFR1/FADD/TRADD axis in cancer cells induce apoptosis and inhibit tumor progression and metastasis. Transl Oncol. 2022;21:101433. 10.1016/j.tranon.2022.101433. 35462210 10.1016/j.tranon.2022.101433 PMC9046888

[133] Dai H, Chen H, Xu J, Zhou J, Shan Z, Yang H, et al. The ubiquitin ligase CHIP modulates cellular behaviors of gastric cancer cells by regulating TRAF2. Cancer Cell Int. 2019;19:132. 10.1186/s12935-019-0832-z. 31130821 10.1186/s12935-019-0832-z PMC6524225

[134] Litchfield K, Reading JL, Puttick C, Thakkar K, Abbosh C, Bentham R, et al. Meta-analysis of tumor- and T cell-intrinsic mechanisms of sensitization to checkpoint inhibition. Cell. 2021;184:596–614.e14. 10.1016/j.cell.2021.01.002. 33508232 10.1016/j.cell.2021.01.002 PMC7933824

[135] Manikandan A, Jeevitha S, Vusa L. Peptidomimetics for CVD screened via TRADD-TRAF2 complex interface assessments. Silico Pharm. 2023;11:28. 10.1007/s40203-023-00166-0. 10.1007/s40203-023-00166-0 PMC10611682 37899969

[136] Vince JE, Chau D, Callus B, Wong WW, Hawkins CJ, Schneider P, et al. TWEAK-FN14 signaling induces lysosomal degradation of a cIAP1-TRAF2 complex to sensitize tumor cells to TNFα. J Cell Biol. 2008;182:171–84. 10.1083/jcb.200801010. 18606850 10.1083/jcb.200801010 PMC2447903

[137] Michaelson JS, Kelly R, Yang L, Zhang X, Wortham K, Joseph IB. The anti-Fn14 antibody BIIB036 inhibits tumor growth in xenografts and patient derived primary tumor models and enhances efficacy of chemotherapeutic agents in multiple xenograft models. Cancer Biol Ther. 2012;13:812–21. 10.4161/cbt.20564. 22669574 10.4161/cbt.20564

[138] Zeng T, Peng L, Chao H, Xi H, Fu B, Wang Y, et al. IRE1α-TRAF2-ASK1 complex-mediated endoplasmic reticulum stress and mitochondrial dysfunction contribute to CXC195-induced apoptosis in human bladder carcinoma T24 cells. Biochem Biophys Res Commun. 2015;460:530–6. 10.1016/j.bbrc.2015.03.064. 25797626 10.1016/j.bbrc.2015.03.064

[139] Lee SU, Sung MH, Ryu HW, Lee J, Kim HS, In HJ, et al. Verproside inhibits TNF-α-induced MUC5AC expression through suppression of the TNF-α/NF-κB pathway in human airway epithelial cells. Cytokine. 2016;77:168–75. 10.1016/j.cyto.2015.08.262. 26318254 10.1016/j.cyto.2015.08.262

[140] Sugano T, Masuda M, Takeshita F, Motoi N, Hirozane T, Goto N, et al. Pharmacological blockage of transforming growth factor-β signalling by a Traf2- and Nck-interacting kinase inhibitor, NCB-0846. Br J Cancer. 2021;124:228–36. 10.1038/s41416-020-01162-3. 33244122 10.1038/s41416-020-01162-3 PMC7782820

[141] Nguyen T, Carrieri FA, Connis N, Lafargue A, Chang J, Chan A, et al. TNIK inhibition sensitizes TNIK-overexpressing lung squamous cell carcinoma to radiotherapy. Mol Cancer Ther. 2024;23:1201–11. 10.1158/1535-7163.MCT-23-0412. 38670554 10.1158/1535-7163.MCT-23-0412 PMC11292318

[142] Zhang R, Yu Y, Yang Y, Zhang M, Zhang X, Chang Y, et al. Therapeutic targeting of TNIK in papillary thyroid carcinoma: a novel approach for tumor growth suppression. Med Oncol. 2024;41:160. 10.1007/s12032-024-02380-y. 38763968 10.1007/s12032-024-02380-y

[143] Zhou K, Cheong JE, Krishnaji ST, Ghalali A, Fu H, Sui L, et al. Inhibition of Wnt signaling in colon cancer cells via an oral drug that facilitates TNIK degradation. Mol Cancer Ther. 2023;22:25–36. 10.1158/1535-7163.MCT-21-0801. 36302395 10.1158/1535-7163.MCT-21-0801

[144] Yao Y, Zhao K, Yu Z, Ren H, Zhao L, Li Z, et al. Wogonoside inhibits invasion and migration through suppressing TRAF2/4 expression in breast cancer. J Exp Clin Cancer Res. 2017;36:103. 10.1186/s13046-017-0574-5. 28774312 10.1186/s13046-017-0574-5 PMC5543547

[145] Fang Y, Liu J, Xin L, Jiang H, Wen J, Li X, et al. Xinfeng capsule inhibits lncRNA NONHSAT227927.1/TRAF2 to alleviate NF-κB-p65-induced immuno-inflammation in ankylosing spondylitis. J Ethnopharmacol. 2024;323:117677. 10.1016/j.jep.2023.117677. 38160870 10.1016/j.jep.2023.117677

[146] Manna SK, Babajan B, Raghavendra PB, Raviprakash N, Sureshkumar C. Inhibiting TRAF2-mediated activation of NF-kappaB facilitates induction of AP-1. J Biol Chem. 2010;285:11617–27. 10.1074/jbc.M109.094961. 20133937 10.1074/jbc.M109.094961 PMC2857039

[147] Song Y, Song B, Yu Z, Li A, Xia L, Zhao Y, et al. Silencing of CPNE1-TRAF2 axis restrains the development of pancreatic cancer. Front Biosci (Landmark Ed. 2023;28:316. 10.31083/j.fbl2811316.. 10.31083/j.fbl2811316 38062811

[148] Kandir S, Karakurt S, Gökçek-Saraç Ç, Karakurt S. Tannic acid elicits differential gene regulation in prostate cancer apoptosis. Acta Pharm. 2024;74:539–50. 10.2478/acph-2024-0020. 39279521 10.2478/acph-2024-0020

